# Can deciphering the growth of Meckel's diverticulum help us to decide the resection technique?

**DOI:** 10.3389/fsurg.2024.1421732

**Published:** 2024-08-30

**Authors:** Mehmet Can, Malik Ergin, Özkan Okur, Ayşe Demet Payza, Kamer Polatdemir, Akgün Oral

**Affiliations:** Dr. Behçet Uz Pediatric Diseases and Surgery Training and Research Hospital, Izmir, Türkiye

**Keywords:** Meckel’s diverticulum, simple diverticulectomy, segmental resection, heterotopic mucosa, wedge resection

## Abstract

**Introduction:**

The employment of laparoscopic surgical techniques has reignited the debate on managing Meckel's Diverticulum (MD) due to its low complication rates. Nevertheless, concerns have been raised regarding completely removing any potential heterotopic mucosa. Our study aimed to compare surgical approaches in MD and assess the effectiveness of simple diverticulectomy.

**Methods:**

Between 2003 and 2022, 139 patients with MD were retrospectively analysed. The study examined the morphometric measurements of the diverticulum and the location of the heterotopic mucosa in the diverticulum regarding growth and symptoms.

**Results:**

Simple diverticulectomy achieved the lowest postoperative complication rate among excision techniques (*p* = 0.03). MD's length, diameter, and distance to the ileocecal valve increase linearly with growth in the first three years of life (*p* = 0.00, *p* = 0.01, *p* = 0.00) but not in subsequent years (*p* = 0.81, *p* = 0.43, *p* = 0.21). As the length of the MD increases, the heterotopic mucosa (HM) is displaced distally (*p* = 0.01). Patients in whom HM reaches the base of the diverticulum always present with bleeding (*p* = 0.02).

**Discussion:**

Simple diverticulectomy is a safe technique for Meckel's diverticulum resection. Meckel's diverticulum continues to grow until the age of 3. With this growth, the heterotopic mucosa is displaced distally and moves away from the base of the diverticulum. Bleeding is the main symptom in patients with HM reaching the base of the diverticulum. In patients with bleeding or younger than three years of age, simple diverticulectomy may not be considered safe.

**Level of Evidence:** III

## Highlights

This is the first study to demonstrate the growth of Meckel's diverticulum and displacement of heterotopic mucosa with age in children. This development may alter surgical preferences for safe diverticulum excision. We believe safe diverticulum excision in children will change the approach to incidental Meckel's diverticulum.

## Introduction

Meckel's diverticulum (MD) is a congenital anomaly of the small intestine resulting from incomplete obliteration of the omphalomesenteric duct (OMD). J.F. Meckel first described it in 1809, but its clinical implications were only appreciated a century later when Salzer and Deetz reported complications associated with the presence of heterotopic mucosa (HM) within the diverticulum. The management of MD is a controversial topic, particularly in cases where it is incidentally detected during surgery and the patient is asymptomatic. While it is widely accepted that symptomatic MDs (SMD) should be surgically removed, the optimal approach for incidental MDs (IMD) is unclear (1, 2). Some surgeons recommend routine resection to prevent future complications and ensure complete removal of HM (3, 4). Some argue for a conservative strategy to avoid unnecessary morbidity and mortality from surgery (1, 5). Laparoscopic techniques have made surgical treatment of Meckel's diverticulum more feasible and less invasive. However, questions have arisen about the adequacy of simple diverticulectomy vs. wedge or segmental resection. Laparoscopic simple diverticulectomy is a faster and more straightforward procedure, but it may leave residual HM in the adjacent bowel wall. On the other hand, wedge or segmental resection is a more radical approach that ensures complete removal of HM. However, it is a technically demanding treatment with a more significant risk of complications in solely laparoscopic operations. Resection can only be performed by removing the ileum outside the abdomen via the umbilical port incision or an open procedure. On the other hand, simple diverticulectomy can be performed with a stapler or just tying off the root, similar to an appendectomy (6). While most surgeons typically select the method based on the clinical presentation or the width or length of the diverticulum (4), the actual efficacy of the chosen technique can only be determined post-pathological examination. A residue might remain after a simple diverticulectomy, or an unnecessary segmental resection might have been performed on a diverticula that does not contain HM. By understanding the rules governing the spread of HM into the diverticulum, we can avoid these pitfalls and make the most appropriate choice.

While some previous studies have hinted at a potential link between the length of the MD and age (7), this connection has not been definitively established due to the absence of neonates and infants in these studies. Furthermore, our study is the first to investigate a shift in the location of HM due to this elongation in medical literature. Our observations show that the MD elongates by age, and the ectopic mucosa moves distally. In light of the observations above, our objective is to examine the requirement for resection in cases of IMD, and the effectiveness of simple diverticulectomy in ensuring the complete excision of heterotopic mucosa on the other.

## Methods

A retrospective analysis was conducted on medical records, operative notes, pathology reports, and specimens of patients treated for MD between 2003 and 2022 at our clinic after the ethics committee decision number 2022/09-01 was obtained from Dr. Behçet Uz Children's Hospital for this article. The study included all patients under 18 years of age diagnosed with MD during surgery, regardless of their primary diagnosis. The study collected demographic data, symptomatic data, and morphometric measurements of MD, including the distance from the ileocecal valve, length, and diameter of the diverticulum. Additionally, histopathologic risk factors, such as HM content, location of HM in the diverticulum, presence of diverticuloperitoneal band, and enterolith, were recorded. Morphometric data was obtained through intraoperative measurements, while patients without these measurements had their data sourced from pathology reports. The follow-up period was calculated based on the date of the last visit after discharge.

The first part of the study assessed the need for resection in IMD patients (*n* = 53) and compared postoperative complication and reoperation rates in SMD patients (*n* = 86) and IMD patients. Resection methods were analysed for postoperative complication rates in MD surgery. These methods included segmental resection (*n* = 66), which involves removing the small bowel segment containing the diverticulum followed by primary small bowel anastomosis; wedge resection (*n* = 33), which entails cutting the base of the diverticulum in a wedge shape to preserve the mesenteric wall and avoid a circumferential suture line; and simple diverticulectomy (*n* = 22), which involves excising the diverticulum by transverse cutting from its base. Laparoscopic completion of resection using an endo stapler is only possible if a simple diverticulectomy is to be performed. On the other hand, wedge and segmental resection can be performed using the classical open surgical method or the laparoscopy-assisted method. In the latter method, the resection is completed manually by taking the MD and adjacent ileal ans out of the abdomen from the umbilical port entry site after the diagnosis is made laparoscopically.

In the second part of the study, morphometric data were analysed according to age to evaluate the adequacy of simple diverticulectomy for total excision of HM. Because HM placement is not routinely reported in pathology reports, all pathology slides were reexamined without the use of previous reports to avoid detection bias and categorised into three groups according to the location of the HM in the MD:

• Tip: HM is limited to the distal half of the diverticulum.
• Middle: HM is present from the apex to the proximal half of the diverticulum but does not reach the base.
• Entire: HM involves the entire diverticulum from the apex to the base of the diverticulum.

The relationship between HM and the growth of the MD was analysed by examining the morphometric data of the MD according to the groups. Statistical analyses included the chi-square test for nominal variables, Pearson correlation for parametric variables, and Kendall Tau B correlation for nonparametric variables. All analyses were performed using IBM SPSS Statistics 24. An *a priori* power analysis was conducted using G-Power version 3.1 to determine the minimum sample size required to test the study hypothesis. Results indicated the sample size needed to achieve 80% power for detecting a 0.5 effect, at a significance criterion of *α* = .05, was *n* = 106 for the Pearson test and *n* = 52 for the Chi-Square test. Thus, the sample size of *n = 139* is adequate to test the study hypothesis.

In our study, missing values were distributed randomly (*p* = 0.399) and assigned using the median of nearby points. A chi-squared test was used to demonstrate the difference in complication rates between IMD and SMD and between resection types. Loess scatter plots were generated based on height, width, and distance from the ICV to show the relationship between MD and age. Since there was a break in the graphs around three years of age, the correlation between diverticulum length, width, and distance to ICV in patients younger and older than three years of age was examined using Spearman's test. Kendall Tau B correlation analysis was used to understand the relationship between the distribution of HM and diverticulum length. In patients where the ectopic tissue covered the entire diverticulum, the difference between presentation with bleeding and other symptoms was shown by chi-squared test.

## Results

From 2003 to 2022, 139 patients were diagnosed with MD in our clinic. The patients were all Caucasian, 106 males and 33 females (M/F = 3.2), with ages ranging from 3 days to 17 years (mean 6.2 years). The average follow-up period of the patients was 26 ± 14 months.

In 53 patients, MD was found incidentally, and 86 patients underwent surgery for symptomatic MD (SMD). While SMD most commonly presents with bleeding, the most common initial diagnosis in patients with IMD is appendicitis (Table 1).

**Table 1:** Surgical presentations of SMD and IMD.

SMD (*n* = 86; 61,9%)	IMD (*n* = 53;39,1%)
Bleeding (*n* = 25, 29.1%)	Appendicitis (*n* = 37;69,8%)
Volvulus (*n* = 19, 22.1%)	Duodenal atresia (*n* = 3; 5,7%)
Invagination (*n* = 16, 18.6%)	Necrotising enterocolitis (*n* = 3; 5,7%)
Perforation (*n* = 14, 16.3%)	Anorectal malformation (*n* = 2; 3,7%)
Diverticulit (*n* = 11, 12.8%)	Umbilical cord hernia (*n* = 2; 3,7%)
Patent OMD (*n* = 1,1.1%)	Gastroesophageal reflux (*n* = 1; 1,9%)
	Esophageal atresia (*n* = 1; 1,9%)
	Burkitt lymphoma (*n* = 1; 1,9%)
	Intestinal injury (*n* = 1; 1,9%)
	Inguinal hernia (*n* = 1; 1,9%)
	Malrotation (*n* = 1;1,9%)

Surgery outcomes for SMD and IMD were compared in terms of postoperative complications and reoperation rates (Table 2). Of 86 SMD patients, 17 (19.8%) had postoperative complications and 7 (8.1%) required reoperation. Of 35 IMD patients, only 3 (8.6%) had postoperative complications, and none required revision. The difference between SMD and IMD was statistically significant for postoperative complications and reoperation rates (*p* = 0.042, *p* = 0.044).

**Table 2:** Complications after MD surgery.

	SMD	IMD	*p* < 0,05
Not Resected	–	Meckel Diverticulitis	
		(*n* = 2) Invagination (*n* = 1)	*p* = 0.01
		Volvulus (*n* = 1)	
Simple Diverticulectomy	Anastomotic leakage (*n*=1)	–	
Wedge Resection	Ileus (*n* = 5)	–	*p* = 0,04
	Anastomotic leakage (*n*=1)		
Segmental Resection	Ileus (*n* = 9)	Ileus (*n* = 2),	
	Intraabdominal abcess(*n* = 1)	Wound infection (*n* = 1)	*p* = 0.04

**Figure 1:**
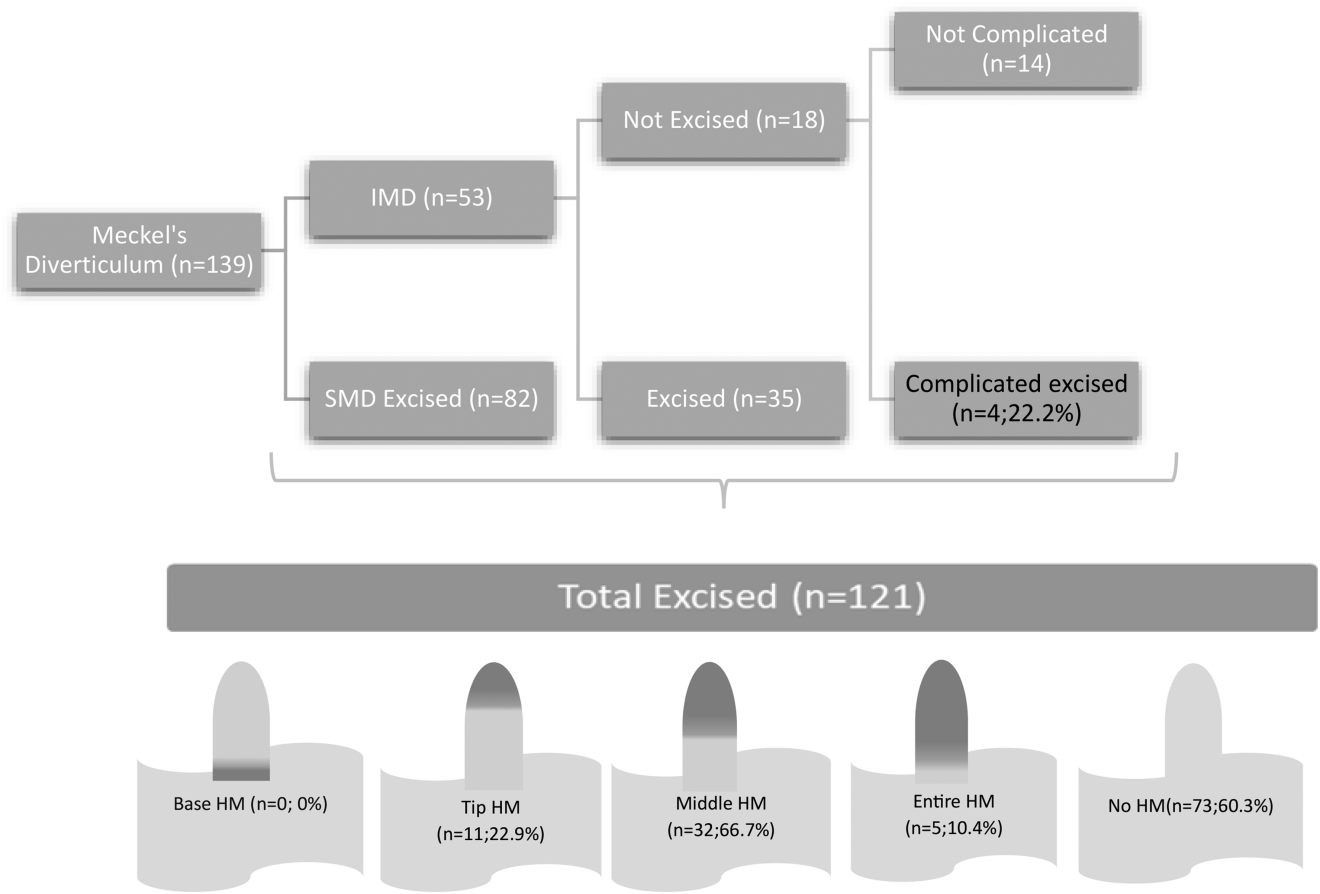
Distribution of HM contained in excised SMD and IMD.

The surgical management of 53 patients with IMD was as follows: 35 (66%) underwent removal of the MD, while 18 (34%) underwent surgery for the primary disease only, and the MD was left intact at the surgeon's discretion because it looked normal. However, four of these patients required reoperation for complications related to the MD (2 diverticulitis, one intussusception, one volvulus) (Figure 1). The complication rate requiring re-operation was 0% in patients who had their IMD removed, compared to 22.2% in patients who did not have their MD removed (*p* = 0.01).

The surgical methods for MD removal included open surgery in 108 cases, laparoscopic surgery in 3 cases, and laparoscopic-assisted surgery in 10 cases. Postoperative complication rates were not significantly different between groups (*p* = 0.49).

Segmental resection was the most common surgical technique (47.5%, *n* = 66), followed by wedge resection (23.7%, *n* = 33) and simple diverticulectomy (15.8%, *n* = 22). The postoperative complication rates were 19.7%, 18.2% and 0%, respectively. Simple diverticulectomy had a significantly lower complication rate than the other procedures (*p* = 0.03).

Of the 90 SMDs resected, 58 (64.4%) had one or more histopathologic risk factors such as enterolith, diverticuloperitoneal band (DPB) or HM. Of the 35 IMDs that were resected, 11 (31.4%) had any histopathologic risk factor. The difference between SMD and IMD was statistically significant (*p* = 0.00). Of the 121 specimens examined, 48 (39.7%) had HM. The most common type of HM was gastric (*n* = 40, 83.3%), followed by pancreatic (*n* = 5, 10.4%). Three patients (6.3%) had both gastric and pancreatic HMs.

**Figure 2:**
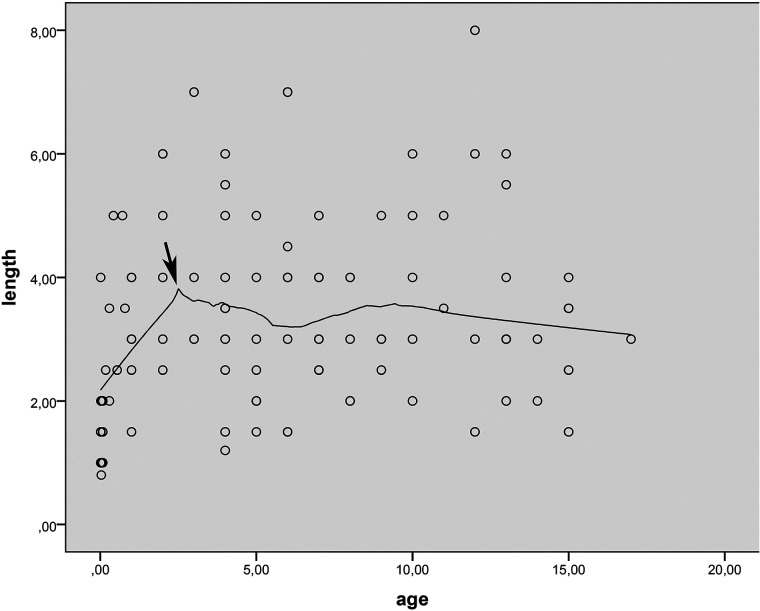
Loess scatter-dot graphs showing the distribution of age-to-length. Arrow marks the point where the MD height stops increasing in proportion to age.

The diverticulum was located at a distance of 10–85 cm (mean 41.7 cm) from the ileocecal valve (ICV). Distance to ICV data for 18 patients could not be obtained and was considered missing. The distance to the ICV showed a linear increase during the first three years of life (*p* = 0.00, r = 0.56) but not after that. The length of the diverticulum ranged from 0.8 to 8 cm (mean 3.4 cm) and the diameter from 0.3 to 3.5 cm (mean 1.6 cm). The length of the diverticulum also increased linearly up to 3 years of age (*p* = 0.00 r = 0.62) but not beyond that age (*p* = 0.81) (Figure 2). The same pattern was observed for diverticulum diameter. It increased with age in the first 3 years (*p* = 0.01, r = 0.43), but not in the following years (*p* = 0.07).

**Figure 3:**
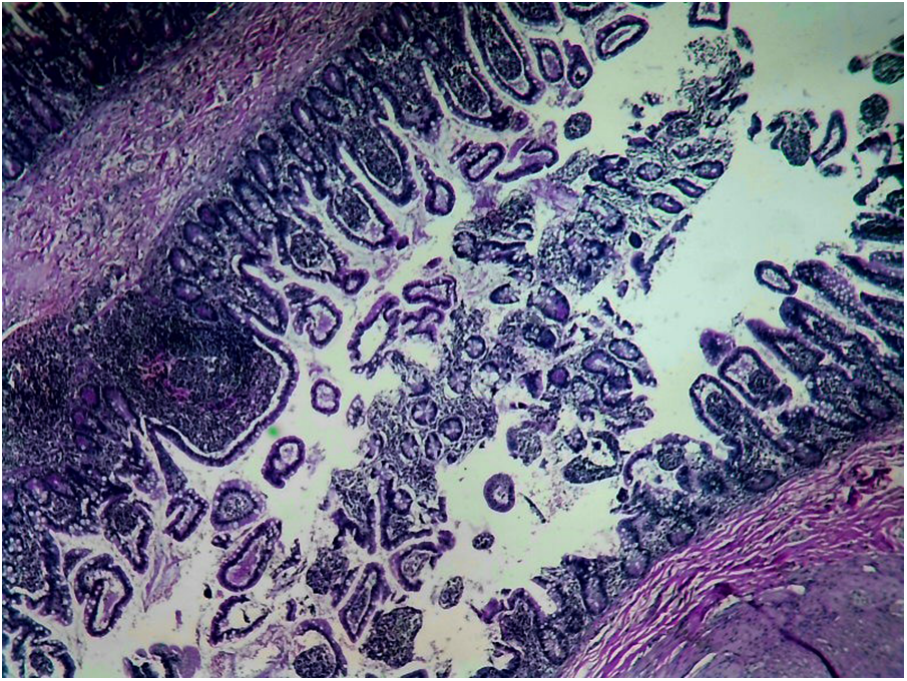
No HM in the diverticulum.

**Figure 4:**
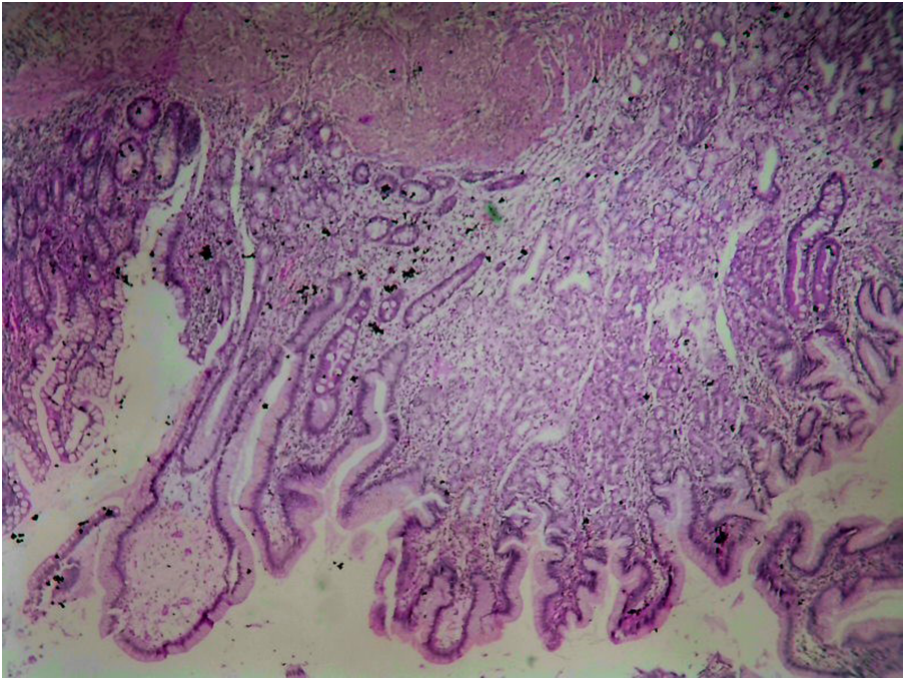
HM reaches to the middle part of the diverticulum.

**Figure 5:**
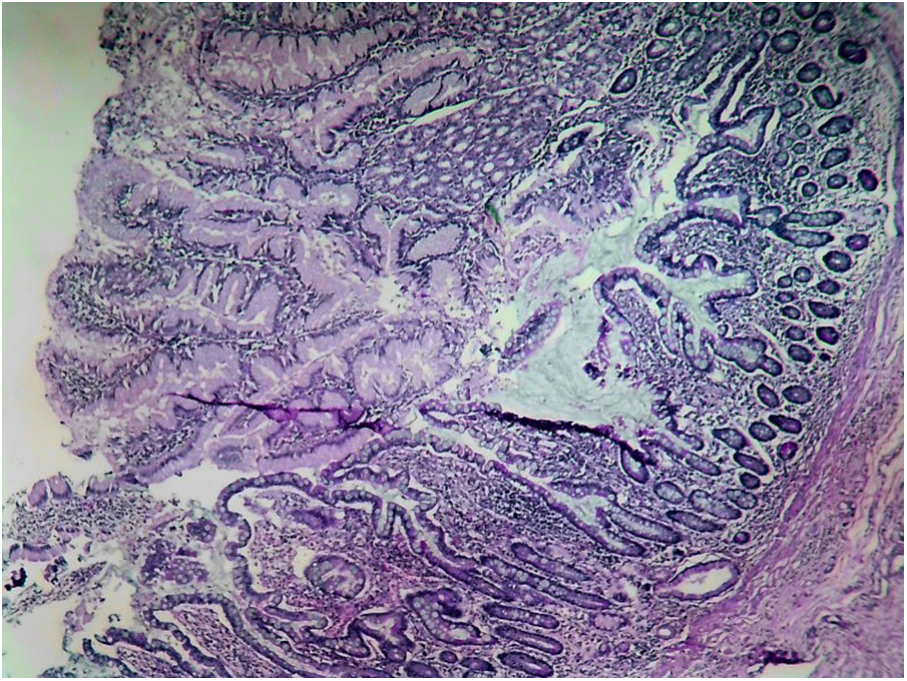
HM reaches the base of the diverticulum.

Of the 121 diverticula removed, 73 (60.3%) showed no evidence of HM (Figure 3). However, 48 (39.7%) had HM in some parts. None of the diverticula had HM only in the basal part without affecting the distal part. The distribution of HM in the diverticulum was tip in 11 cases (22.9%), mid in 32 cases (66.7%) (Figure 4), and Entire in 5 cases (10.4%) (Figure 5).

**Figure 6:**
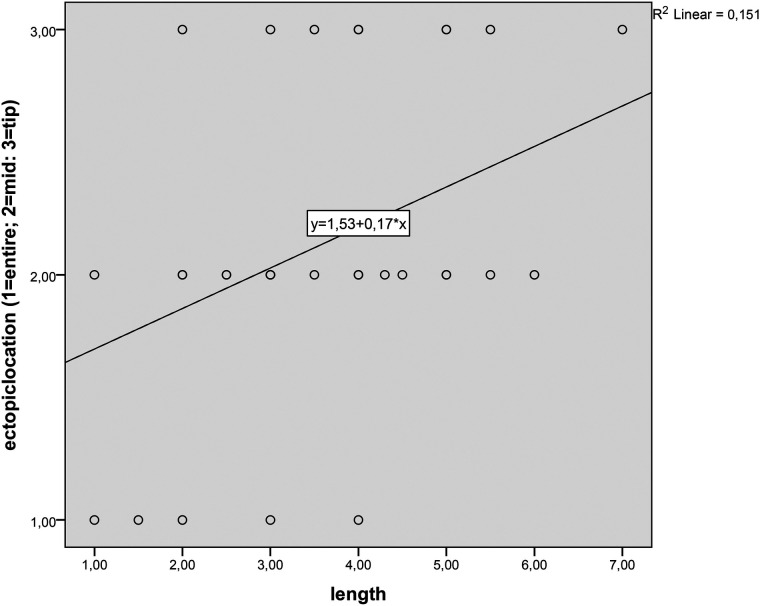
Linear scatter-dot graphs of length to the ectopic tissue location of MD.

The location of the HM in the diverticulum is linearly related to its length (Figure 6), meaning that as the length of the diverticulum increases, the HM moves distally from the base of the diverticulum (*p* = 0.01, r = 0.31). However, the width of the diverticulum does not affect the position of the HM (*p* = 0.95).

Four of the five patients with HM involving the entire diverticulum, including the base of the diverticulum, had bleeding and one had pOMD. HM did not extend to the base of the diverticulum in patients with other complications. There is a significant correlation (*p* = 0.025) between HM extension to the base of the diverticulum, bleeding and pOMD.

## Discussion

The MD is a vestigial structure that arises from the omphalomesenteric duct (OMD) and connects the embryonic midgut to the yolk sac. It is located on the opposite side of the mesentery in the last part of the ileum, 7–200 cm before the ICV. Its length and diameter range from 0.4 to 11 cm and 0.3 to 7 cm, respectively (2, 8). It is more common in males, with a ratio of 1.5–4 (2–4, 6). This ratio is consistent in children, as shown in our study.

MD-associated complications include diverticulitis, bleeding, volvulus, invagination, umbilical anomalies, and tumour development (2, 3, 6, 8). Our study found that diverticulitis and bleeding were the most common complications, consistent with the literature (2). None of our patients developed tumours.

SMD requires surgical removal. The best management of IMD found during surgery for another reason is not clear. In our study, most cases of IMD (70.6%) occurred during surgery for suspected acute appendicitis. In IMD, the surgeon must make a quick decision whether to remove the MD prophylactically or not, as it is usually not possible to check the surgical margin for ectopic tissue. Some argue against prophylactic removal, citing the low risk of complications in asymptomatic MD and the fact that this risk decreases with age (1, 5). Others, however, are reluctant to leave IMD unremoved, considering that up to 20% of MDs have HM and that removal is more risky if complications occur (3, 4). Furthermore, HM is not the only factor that increases the risk of MD. In our study, 31.4% of IMDs had one or more risk factors (HM, DPB, enterolith). In addition, studies against prophylactic removal did not evaluate the risk of complications from IMD left in the abdomen with surgical adhesions. In our series, the complication rate of unexcised IMD was 22.2%. Cullen reported a lower risk of 6.4% in people under 80 years of age but still advised prophylactic removal, considering this risk to be high (9).

Some people argue that prophylactic resection is risky because of the high rate of surgical complications in patients who undergo resection (1). However, with improved surgical techniques, complication rates have been reported to be lower. Our study found that postoperative complications and the need for reoperation were significantly lower after IMD surgery than after SMD surgery. Some studies reported no complications after IMD resection (10). This may be due to the widespread use of laparoscopic surgery, which has a significantly lower complication rate than MD (11). Laparoscopic or laparoscopic-assisted resection of MD has been recognised as a safe and effective method, and it has been suggested that MD detected by diagnostic laparoscopy should be resected laparoscopically instead of converting to laparotomy (2, 12). Our study found no significant difference in postoperative complications among patients who underwent laparoscopic, laparoscopic-assisted, and open-surgical methods. This may be because we have yet to reach the learning curve, as shown by the few patients who underwent the laparoscopic procedure.

The management of incidental Meckel's diverticulum (IMD) in adults is still controversial, but the presence of IMD in children is considered a risk factor (4, 13, 14). Our study showed that IMD in children is associated with a high risk of complications and should be surgically removed. In addition, we found that the removal of IMD has fewer postoperative problems than the removal of SMD, which supports the resection of IMD in children.

There are three possible methods for intraoperative removal of the diverticulum: segmental resection, wedge resection, or simple diverticulectomy. Simple diverticulectomy has become more familiar with the advent of laparoscopic procedures because it is easier to perform. Our study showed no patients who underwent simple diverticulectomy (*n* = 22) had any postoperative complications. However, the effectiveness of simple diverticulectomy in removing all HM within the MD is doubtful. Mukai observed that HM in the MD extends from distal to proximal; he only found HM in the proximal part with being in the distal part (15). Based on the embryological theory, since the gastric mucosa does not originate from the midgut, HM differentiates from multipotent cells in the part of the OMD that is distally connected to the yolk sac and, therefore, spreads into the diverticulum from the distal part (3, 15). We did not find HM only in the base of the diverticulum without being in the distal part in any of our patients, which supports the embryologic theory. Our study also showed that during the first three years of life, the MD grows longer and broader and moves away from the ICV. As the MD increases, the HM is pushed distally and moves away from the base of the diverticulum. Our results indicate that MD growth is related to age, confirming Gezer's suggestion that length is related to age (7) but that this growth stops around three years.

The relationship between the HM's location and the MD's length is known, but no morphologic parameter can predict it (16). Vercoe proposed that the HM is located in the head or body of the diverticulum when the length/diameter ratio is greater than two and in the base when it is less than 2 (3). Mukai suggested a ratio of 1.6 instead (15). Robijn and Park identified a diverticulum length of more than 2 cm as a risk factor (4, 13). Slivova and Sinopidis recommended excision of diverticula with diameters greater than 1.5 cm and 2.5 cm, respectively, as they may contain HM (17, 18). The different morphological descriptions are due to the various age groups in the studies. Our results show that the growth of the length and diameter of the MD stops after three years of age, so the morphometric values vary depending on the average age of the study groups. Therefore, the patient's age should be considered more than the morphometric measurements of the diverticulum. Simple diverticulectomy may be risky in the first three years because possible HM content may not have migrated distally.

Simple diverticulectomy, which Glenn advocates for patients with bleeding, is widely considered to be unsafe (16, 19–22). Our histopathologic analysis showed that patients with HM extending to the base of the diverticulum always present with bleeding or pOMD. The ulcers that cause bleeding are located in the intestinal mucosa near the HM and often in the diverticulum neck, entirely covered by the gastric mucosa (23). Thus, in bleeding or pOMD patients, the HM may spread to the base of the diverticulum, and simple diverticulectomy may not remove all of the HM.

A limitation of the study is that a clear age-cut-off value for diverticulum extension could not be determined, despite the Loess Scatter dot plot indicating that it continued until around three years of age. Additionally, the number of diverticula containing HM was small. A higher correlation coefficient between the lengthening of the diverticulum and the distal displacement of the HM can be demonstrated in a more extensive series.

To improve surgical outcomes, we need to rethink how we treat IMD. Many children with IMD have risk factors, and complications can arise if IMD is not removed. Therefore, removing IMD is better than leaving it inside the abdomen because it has a shallow risk of complications. Cutting off MD with simple diverticulectomy is becoming more common in laparoscopic surgery because it is easy to do. However, there is little evidence in the literature that this procedure works well for removing HM. Our study shows that MD grows until the child is three years old, and HM moves further away from the diverticulum. In younger patients, HM may not be far enough from the border of the diverticulum. Also, based on our findings, patients who have HM reaching the border of the diverticulum always have bleeding. Therefore, simple diverticulectomy may not remove all of HM in patients younger than three years old or in patients who have bleeding or pOMD.

Our study's findings are likely to positively impact the treatment of IMD in children. Due to the insights gained from our research, we hope this congenital intestinal anomaly will no longer be left untreated. Additionally, our study may help us better understand the limitations of simple diverticulectomy, which has become increasingly popular with the advancement of laparoscopic techniques.

## Data Availability

The datasets presented in this study can be found in online repositories. The names of the repository/repositories and accession number(s) can be found below: http://doi.org/10.6084/m9.figshare.25563141.
